# Clinical Evidence of Effects of Green Mandarin (Putgyul) Extract on Skin Aging: A Randomized, Double Blind, Placebo-Controlled Study

**DOI:** 10.3390/nu14071352

**Published:** 2022-03-24

**Authors:** Young-Min Ham, Seon-A Yoon, Hyejin Hyeon, Ho-Bong Hyun, Sung-Chun Kim, Boram Go, Yong-Hwan Jung, Weon-Jong Yoon

**Affiliations:** Biodiversity Research Institute, Jeju Technopark, Jeju 63608, Korea; hijel@jejutp.or.kr (Y.-M.H.); yoonsa33@jejutp.or.kr (S.-A.Y.); hhj2065@jejutp.or.kr (H.H.); hyebong@jejutp.or.kr (H.-B.H.); sckim@jejutp.or.kr (S.-C.K.); boram01@jejutp.or.kr (B.G.); yhjung@jejutp.or.kr (Y.-H.J.)

**Keywords:** *Citrus unshiu* Marcov., Putgyul extract, skin aging, narirutin, functional food

## Abstract

Green mandarins are widely consumed unripe as mandarin oranges (*Citrus unshiu Marcov.*), which exhibit anti-inflammatory and anti-wrinkle effects by inhibiting the production of inflammatory cytokines and matrix metalloproteinase. A randomized, double-blind, placebo-controlled clinical study was performed to verify the skin improvement efficacy and safety of green mandarin extract (PTE). For the standardization of PTE, narirutin was set as a marker compound, and PTE with a constant narirutin content was prepared for the study. After randomizing subjects with periorbital wrinkles, they were orally administered PTE (300 mg/day) or a placebo for 12 weeks. Periorbital wrinkles were measured using PRIMOS^CR^ SF. Skin elasticity, moisture content, transepidermal water loss, and gloss were also measured. In the study results, the depth, volume, and skin roughness of the periorbital wrinkles were significantly improved compared to the control group (*p* = 0.011, 0.009, and 0.004, respectively). The survey confirmed that the skin condition improved after PTE consumption for 12 weeks. No adverse reactions associated with PTE were observed during the study period. Thus, the results demonstrate that PTE effectively improves UV-induced skin wrinkles. Therefore, it is considered that PTE has sufficient value as a functional food ingredient that can prevent skin aging.

## 1. Introduction

Among the various aging phenomena of the human body, skin aging constitutes the most sensitive aging phenomenon because it can be easily recognized.

Skin aging can be divided into intrinsic and extrinsic factors. Extrinsic factors include various environmental factors, such as ultraviolet radiation (UV), cold, fine dust, and stress. Among them, UV is one of the main causes, known as photoaging. UV is classified into UVA (315–400 nm), UVB (280–315 nm), and UVC (100–280 nm), according to the wavelength. Unlike UVC, which is mostly absorbed in the ozone layer, UVB reaches the surface and penetrates to the epidermis, whereas UVA penetrates to the dermis, and both play important roles in photoaging [1,2].

DNA damage, oxidative damage to proteins and lipids, and inflammation occur in skin exposed to UV, by activation of skin barrier damage, generation of reactive oxygen species, and pro-inflammatory cytokines [3,4,5]. Accordingly, the production of collagen, elastin, hyaluronic acid, fibrillin, etc. in the extracellular matrix of the dermal layer decreases, and the biosynthesis of matrix metalloproteinases (MMPs), an enzyme that decomposes collagen, increases, thereby accelerating the aging rate, which induces thick and deep wrinkles, loss of elasticity, dryness, and pigmentation [6,7,8].

Because the skin is continuously aging due to repeated UV exposure, research on healthy functional foods that can prevent the above skin damage through daily intake is sought after [9,10].

In particular, natural plant-derived substances ingested as food have been proven to be safe through long-term ingestion experience, are rich in phytochemical components, such as flavonoids, polyphenols, vitamins, and alkaloids, and are known to have an excellent antioxidant activity [11,12]. Therefore, scientific verification of the effects of natural products on skin aging is essential at the level of clinical trials.

According to a recent report, it was confirmed that fermented honeybush extract [13] and astaxanthin [14] were safe for the human body and improved skin aging. Fermented honeybush extract downregulates MMPs by inhibiting the expression of inflammatory mediators induced by UVB exposure and inhibiting the activity of mitogen-activated protein kinase (MAPK) [13]. Ingestion of astaxanthin increased the minimum erythema dose (MED) and improved the roughness of the skin damaged by UV rays [14]. The administration of astaxanthin also prevented the in-vivo UV-induced production of lipid peroxide and upregulation of ROS-producing enzymes, xanthine oxidase and NADPH oxidase 4 [15]. Furthermore, the administration of astaxanthin prevented the UV-induced decrease in the expression of endogenous antioxidant enzymes, such as superoxide dismutase and glutathione peroxidase [15]. It improves the skin damage caused by UVB by helping the antioxidant mechanism in the body.

Green mandarins (Putgyul) are unripe mandarin oranges (*Citrus unshiu* Marcov.), cultivated in large quantities on Jeju Island, Korea. Mandarin oranges contain vitamins, organic acids, minerals, and flavonoids [3,16]. Moreover, unripe green mandarins, contain about 30% more flavonoids, such as narirutin and hesperidin, than fully ripe fruit [17].

In our previous study, 50% ethanol extract (Putgyul extract, PTE) from green mandarins showed an increase in collagen production by inhibiting matrix metalloproteinase (MMP)-1 in a concentration-dependent manner in human dermal fibroblast (HDF) cells. In addition, PTE intake decreased the expression of inflammatory cytokines and MMP-2 in a concentration-dependent manner in hairless mice with UVB-induced skin damage. Furthermore, the increase in epidermal thickness, β-glucosidase, and collagen fibers, and decrease in transdermal water loss (TEWL), were also confirmed [18].

Therefore, based on these in-vivo results, a clinical study was designed to verify the efficacy and safety of PTE as a functional health food in this study. It was hypothesized that PTE administration (standardized dose) for 12 weeks, to subjects with UV-induced periorbital wrinkles, would lead to an improvement in the depth, volume, length, and roughness of the periorbital wrinkles, as the primary efficacy endpoint. Furthermore, the study was conducted after designing a clinical study with secondary efficacy endpoints, including a survey on skin moisture content, TEWL, skin elasticity, skin gloss, and product efficacy.

## 2. Materials and Methods

### 2.1. Plant Material

In August 2020, one ton of green mandarins were purchased from a farmer in Jeju, Korea. They were then washed, followed by pulverization and freeze-drying (11.4% yield). Green mandarin lyophilisate (50 kg) was extracted with 50% ethanol while stirring at 25 ± 5 °C for 24 h, then filtered, and concentrated under reduced pressure. The concentrate was lyophilized to obtain 50% ethanol extract powders of green mandarins (PTE, 10 kg, 20% yield).

The content of narirutin (naringenin-7-O-rutinoside), a marker compound of PTE, was analyzed using high-performance liquid chromatography (Alliance, Waters, Milford, MA, USA). A photodiode array (Alliance, Waters, Milford, MA, USA) was used as a detector. A zorbox SB-C18 (4.6 mm × 250 mm, 5 µm, Agilent) was used as a column. The flow rate of the mobile phase was 1.0 mL/min and the column temperature was maintained at 30 °C. Distilled water (A) and acetonitrile (B) containing 0.5% acetic acid were mobile phases. Solvent A was lowered from the initial condition of 90% (90/10, *v*/*v*) to 80% (80/20, *v*/*v*) for 70 min and then eluted at 50% (50/50, *v*/*v*) until 75 min. The sample injection volume was 10 µL, and the detection wavelength was 266 nm.

Test material was prepared by mixing with dextrin and crystalline cellulose to ingest 300 mg of PTE by taking two tablets daily. Placebo was prepared with the same ingredients except for PTE. Food coloring and citrus flavor were mixed to match the taste, color, and flavor of the test material.

### 2.2. Subjects

**Figure 1:**
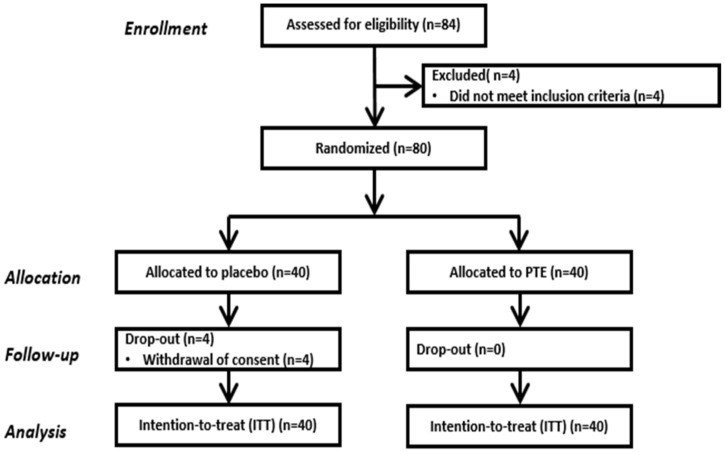
Study flow chart. Flow diagram of the progress though the phase of a parallel randomized trial of two groups (enrollment, allocation, follow-up, completion and data analysis).

Subjects were recruited by posting a notice approved by the IRB on the website of the clinical trial institution, or by attaching posters inside and outside the clinical trial institution. Subjects with all of the following criteria were selected as study participants: women aged 40 or older but less than 60, dry skin with less than 48 A.U. (corneometer value), and those with periorbital wrinkles (grade 3 or higher). We used the global skin wrinkle grade, which evaluated wrinkles on a scale of zero to nine, referring to the ‘Guideline for Efficacy Evaluation of Functional Cosmetics’ published by the Korean Ministry of Food and Drug Safety. In addition, if at least one of the following criteria was met, subjects were excluded from the study: (a) irritation or allergy reported to ingredients related to the test material, (b) if topical dermatological drugs containing steroids were used within 6 months of the commencement of the study, (c) if oral retinoids/steroids were taken to treat wrinkles and moisturizing effects within 3 months of the start of the study, (d) consumption of functional foods for improving gloss and elasticity if the test site was treated within 3 months of the beginning of the study, (e) if the duration was less than 3 months since participating in the same study, (f) in case of a disease that could potentially affect the study, (g) in case of skin disease at the study site, (h) in case of sensitivity or hypersensitivity of the skin, and (i) in case of skin abnormalities such as spots, acne, erythema, and telangiectasia at the study site. After a total of 84 people were recruited, 80 people were enrolled by screening for suitability of study subjects (Figure 1).

### 2.3. Study Design

This study was a randomized, double-blind, placebo-controlled clinical trial. The study was performed as per the Declaration of Helsinki and the International Conference on Harmonization Guidelines for Good Clinical Practice. The study protocol was approved by the H&Bio SRC Institutional Review Board (IRB No. HBABN01-200814-HR-0073-02). Written consent was obtained in advance from all subjects. After suitability screening, the study subjects were randomly assigned to either the control group or the PTE intake experimental group. Subjects took the test material for 12 weeks. Participants were assigned to the regimens at a 1:1 ratio using a computer-generated randomization schedule. We used stratified block randomization to achieve balance among the groups using a block size of 2~8. Both study subjects and investigators were kept double-blind. Each study subject assignment code was sealed and stored in a single opaque envelope, which was designed to be opened only in a medical emergency. A total of 5 visits were scheduled: at week 3 (screening), week 0 (randomization and baseline measurement), week 4, week 8, and week 12.

During the 12 weeks, study subjects consumed placebo or test material containing PTE by taking two tablets per day. Test material was provided every 4 weeks. The Test material and container received at the previous visit of study subjects were checked, and the remaining quantity was recorded.

### 2.4. Efficacy and Safety Assessment

Primary and secondary efficacy and safety evaluation items were measured before intake, and at weeks 4, 8, and 12 after intake. After cleaning the study area, efficacy was evaluated after resting for 30 min under constant temperature and humidity (22 ± 2 °C and 50 ± 5%).

The primary efficacy endpoint of periorbital wrinkles was measured using a three-dimensional skin measurement device PRIMOS^CR^ SF (Canfield, NJ, USA) by analyzing wrinkle parameters (1. average depth of wrinkles, µm, 2. mean depth of biggest wrinkles, µm, 3. max. depth of biggest wrinkle, µm, 4. total wrinkle area, mm, 5. total wrinkle volume, mm, 6. total length of wrinkles, mm, 7. Ra (surface roughness), µm). The entire face was photographed in optical and polarized mode using VISIA^®^ CR (Canfield, NJ, USA) to use as supporting data for visual evaluation of periorbital wrinkles. Three face pictures were taken at each visit. Skin moisture content was measured on the cheeks using Corneometer^®^ CM 825 (Courage + Khazaka electronic GmbH, Cologne, Germany). The moisture content was measured three times and the average was calculated. For TEWL, the left or right cheek area was measured using Tewameter TM300 (Courage + Khazaka electronic GmbH, Cologne, Germany). TEWL was measured three times and the average was calculated. Skin elasticity was measured using Cutometer^®^ MPA580 (Courage + Khazaka electronic GmbH, Cologne, Germany) on the left or right cheek area, and then R2 parameters were analyzed. The R2 parameter is a parameter representing the overall elasticity as the rate at which the state of maximally stretching the skin using the pressure zone recovers to its original state. Skin gloss was measured on the left or right cheek area using a Spectrophotometer^®^ CM26dG (Minolta, Japan). Skin gloss was measured three times and the average was calculated. The questionnaire evaluation on the efficacy of the test material was scored on a scale of 1 to 5 at weeks 4, 8, and 12 post-intake (1 point: very dissatisfied; 2 points: somewhat dissatisfied; 3 points: no change; 4 points: somewhat satisfied; and 5 points: very satisfied). A total of five items were surveyed: ‘skin moisturization’, ‘increase in skin gloss’, ‘skin firmness’, ‘reduction in periorbital wrinkles’, and ‘improvement in skin condition’. For safety assessments, vital sign measurements (blood pressure, pulse beat, and body temperature), the routine battery of blood (complete blood cell count, differential white blood cell count, hemoglobin, hematocrit, platelets, aspartate aminotransferase, alanine aminotransferase, gamma glutamic transpeptidase, triglyceride, high-density lipoprotein, low-density lipoprotein, blood urea nitrogen, creatinine) and urine tests, and adverse event monitoring were conducted.

### 2.5. Statistical Analysis

For the comparison between the two groups, the difference in the mean change between the experimental and control groups was evaluated as a two-tailed test using a 95% confidence interval and *p*-values less than 0.05 on a two tailed test were considered statistically significant. When analyzing the periorbital wrinkle evaluation index, all scores observed in this clinical study were based on descriptive statistics (mean, standard deviation, median, minimum, maximum) for changes at week 12 compared to the baseline. When normality was satisfied, the paired *t*-test and two-sample *t*-test were used to determine the significance of differences within groups and between groups, respectively. However, data were analyzed using the Wilcoxon signed-rank test and Wilcoxon rank-sum test for comparisons within groups and between two groups, respectively, when the normality assumption was violated. In addition, when analyzing periorbital wrinkles, skin moisture content, TEWL, skin elasticity, skin gloss, and questionnaire evaluation index, descriptive statistics (mean, standard deviation, median, minimum, maximum) for all visit time-points were presented, and repeated-measure ANOVA was used to verify the comparison between time points and groups.

## 3. Results

### 3.1. Analysis of Marker Compound Content in PTE

**Figure 2:**
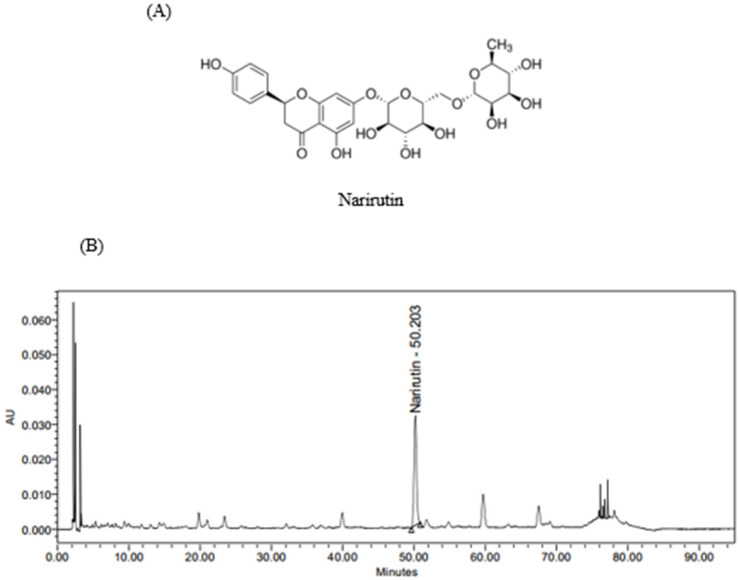
Active compound contained in PTE (**A**) and a HPLC chromatogram (**B**) of narirutin from PTE.

It has been reported that green mandarins contain various flavonoid components. This study selected narirutin (Figure 2A) as a marker compound for PTE, among various components. In the HPLC analysis, narirutin was detected in PTE at 50.2 min, and the content of narirutin was analyzed to be 40.07 ± 0.46 mg/g. The HPLC chromatogram is shown in Figure 2B.

### 3.2. Baseline Characteristics

Among the eighty subjects who were randomly assigned to the two groups, four subjects withdrew (from the placebo group) during the intervention period because of private reasons (Figure 1). The subjects were all women and their average age in the experimental and control group was 47.18 ± 4.402 and 46.23 ± 3.977 years, respectively. The baseline characteristics of the subjects are shown in Table 1. There was no significant difference in BMI and blood pressure between the two groups. Furthermore, there were no clinically significant changes in vital signs, complete blood count, serum biochemistry examination, and urinalysis during the 12-week intake period.

### 3.3. Primary Efficacy Endpoint: Changes in Eye Wrinkles

**Figure 3:**
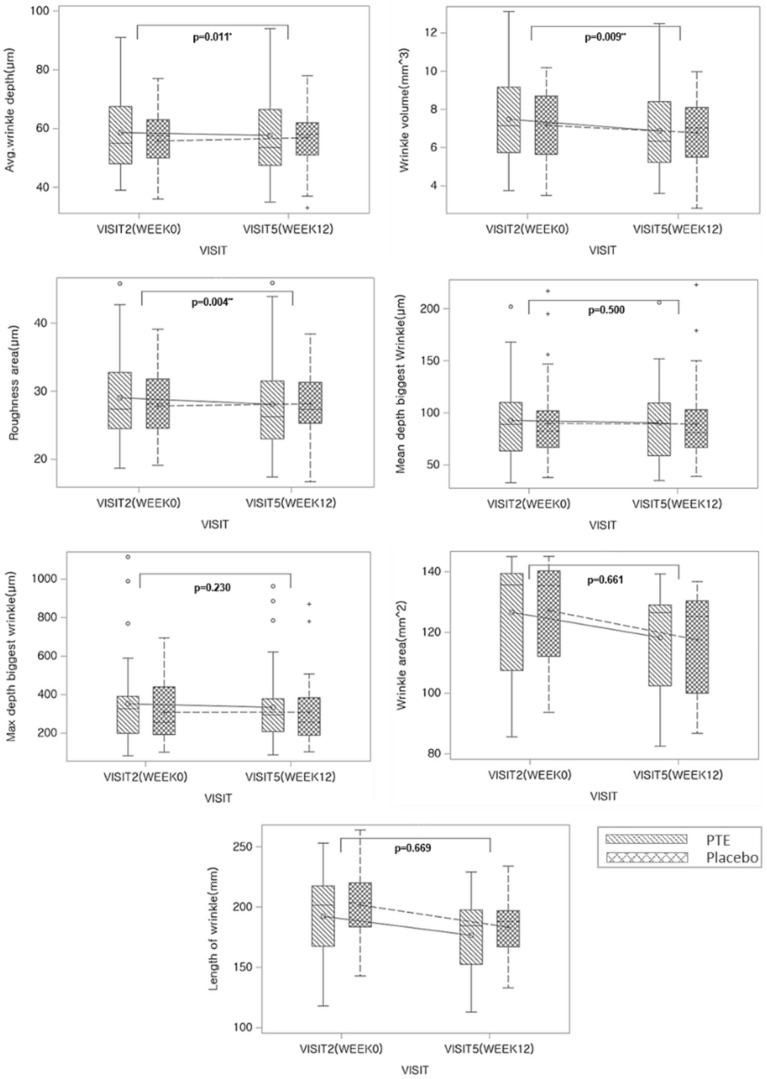
Change from baseline values of skin wrinkles on the crow’s feet during PTE treatment. *p*-value by independent *t*-test; * *p* < 0.05, ** *p* < 0.005.

The change in eye wrinkles was analyzed as the change in measured values of eye wrinkles at week 0 and 12 between the groups. As a result of the average depth of periorbital wrinkle analysis, there was a significant difference between the changes in the experi-mental group and the changes in the control group (*p* = 0.011). In addition, as a result of analyzing the volume of periorbital wrinkles (*p* = 0.009) and skin surface roughness (*p* = 0.004), there were significant differences in the amount of change between the experimental and the control group. However, the changes in the mean depth biggest wrinkle (*p* = 0.500), maximum depth biggest wrinkle (*p* = 0.230), wrinkle area (*p* = 0.661), and length of wrinkle (*p* = 0.669) were not significant (Figure 3 and Table 2).

### 3.4. Secondary Efficacy Endpoints

Secondary efficacy endpoints consist of indicators of secondary skin improvement, affected by PTE intake and estimated by survey evaluation. There was no significant difference between the groups in skin moisture content, TEWL, skin elasticity, and skin gloss (Table 3). In the survey evaluation, there was a significant difference between the time points in ‘skin became moist’ by analyzing the questionnaire on the efficacy of the product (*p* = 0.001), but there was no interaction between groups and time points. Similarly, there was a significant difference between time points in ‘skin became glossier’ (*p* = 0.001), but there was no interaction between the groups and time points. Next, there was a significant difference between the time points in ‘skin became firmer’ (*p* = 0.001), but there was no interaction because it was not significant between groups and time points. However, a significant difference was found between groups at week 8 (*p* = 0.018). It was also confirmed from the subjective survey results that the experimental group had firmer skin at week 8 than at week 4 compared to the control group. Next, there was a significant difference between time points (*p* = 0.001) and between time points and groups (*p* = 0.025) in “periorbital wrinkles were reduced“, confirming the interaction between groups and time points. By performing the *t*-test (total 3 times) between the two groups, at each time point, a significant difference was found at week 12 (*p* = 0.040). It was also confirmed from the subjective survey results that the periorbital wrinkles decreased more in the experimental group than in the control group at week 12 than at week 4 or week 8. Finally, there was a significant difference between the time points in ‘the skin condition was overall improved’ (*p* = 0.001). There was no interaction between groups and time points, but significant differences were confirmed at week 12 (*p* = 0.021). It was also confirmed from the subjective survey results that the skin condition of the experimental group was improved significantly at week 12 more than at week 4 or week 8 compared to the control group (Table 4).

## 4. Discussion

The skin serves as the bulwark between the body and the environment, and is the largest organ of the human body [19]. The skin is subjected not only to internal aging processes, but also to various external stressors, which, in concert, lead to distinct structural changes that influence not only the skin’s appearance, but also its various physiological functions [19]. Skin thickens over the first 20 years of life and then begins to thin progressively, at a rate that accelerates with age [19]. The epidermis, specifically, decreases in thickness with age when unexposed epidermal skin thins by up to 50% between the age of 30 and 80 [19]. The main cause of reduced skin thickness in aging adults is the loss of skin collagen and elastin [19]. In addition, the moisture content of aged skin, particularly that of the stratum corneum, is lower than that of younger skin [19]. Particularly, the moisture content of the stratum corneum decreases progressively with age, eventually dropping below the level necessary for effective desquamation [19]. Thus, skin care products that delay or defend against skin aging processes are being developed. In addition, many studies have been conducted on medical procedures or cosmetics development to delay or prevent skin aging. Recently, with the growing interest of consumers in functional foods, the number of studies on skin-functioning foods has increased [20,21]. Therefore, in this study, we investigated the efficacy of functional foods containing immature citrus extract on the improvement of skin wrinkles and dermal moisture, gloss, elasticity, and density in elderly women who had started to develop wrinkles or already had wrinkles.

*Citrus unshiu* Marcov. is a fruit-bearing tree that has been cultivated for many years in Korea and Japan. Genus Citrus contains various polyphenols, such as flavanones, flavones, polymethoxylated flavones, flavonols, anthocyanins, etc., [22,23,24]. One of these flavanone-7-O-glycosides, narirutin, is a marker compound of the green mandarin extract. Narirutin has antioxidative [25], antidiabetic [26], anti-inflammatory [27], anti-allergic [28], melanin production inhibition [29], and UV-protection effects [30,31]. In addition, the content of bioactive flavonoids, such as hesperidin, neohesperidin, narirutin, and quercetagenin, in the fruits of genus citrus, vary according to the degree of maturity. The content is very high when it is immature [32]. Therefore, based on the research results, we focused on the potential of green mandarin extract, which is rich in narirutin, as a food ingredient for effective anti-aging.

The production of MMP-1 was decreased, and the synthesis of procollagen type I carboxy-terminal peptide was increased in human dermal fibroblasts by the green mandarin extract. In addition, when the green mandarin extract was ingested for 10 weeks in mice, with skin damage induced by UVB, gene expressions of IL-1β, IL-6, COX-2, iNOS, and TNF-α, including MMP-2, MMP-9, and inflammatory cytokines, were significantly suppressed. In addition, the depth of skin wrinkles was improved, and epidermal thickness, collagen degradation, and transdermal water loss were decreased [18]. Therefore, we conducted a randomized, double-blind, placebo-controlled clinical study to develop green mandarin extract as a functional food ingredient that can improve UV-induced skin aging.

When 300 mg of PTE was taken daily for 12 weeks, the depth and volume of periorbital wrinkles and roughness of the skin were significantly improved. In addition, it was confirmed that the overall skin condition was improved through the survey results obtained from the study subjects.

Even though the fundamental mechanism by which wrinkles and roughness of the skin were improved by PTE was not elucidated, on the basis of previous in-vitro and in-vivo findings, we speculate that green mandarin intake affects the reduction in collagen degradation. In addition, it was presumed that polyphenols, which are abundantly contained in green mandarins and have previously been reported to have antioxidant and anti-inflammatory effects, helped improve the skin damage caused by UV.

Moreover, previous studies highlighted that Citrus fruits contain large amounts of flavonoids, along with their various biological activities. Interestingly, it has been reported that flavonoid contents change during the maturation of Citrus, and immature Citrus are more enriched in flavonoids, such as hesperidin, neohesperidin, narirutin, and quercetagetin than mature Citrus. Specifically, flavonoid components of immature Citrus have been reported to prevent photoaging and anti-inflammatory activities in human dermal fibroblast and keratinocytes. In addition, it has been reported, based on UVB-induced skin cells and animal models, that hesperidin and narirutin shield human keratinocytes. Consequently, flavonoids, hesperidin, and narirutin in immature Citrus are believed to reduce skin wrinkles and improve skin moisture. The value of PTE as a functional food ingredient that can help improve skin damaged by UV irradiation was confirmed through this study.

## Figures and Tables

**Table 1:** Baseline characteristics ^A^.

	Placebo (*n* = 40)	PTE (*n* = 40)	*p*-Value ^B^
Gender (N, male/female)	0/40	0/40	-
Age (years)	46.2 ± 3.9	47.1 ± 4.4	0.314
BMI (kg/m)	23.5 ± 2.8	22.7 ± 3.0	0.244
SBP (mmHg)	113.1 ± 13.1	111.1 ± 13.4	0.519
DBP (mmHg)	78.5 ± 5	75.2 ± 11.2	0.144

^A^ Mean ± standard error of the mean. BMI, body mass index; SBP, systolic blood pressure; DBP, diastolic blood pressure. ^B^ *p*-value by independent *t*-test.

**Table 2:** Change in wrinkle parameters measured by PRIMOS^CR^ SF (Canfield, NJ, USA).

Parameter	Visit	PTE (*n* = 40)			Placebo (*n* = 40)			# *p*	## *p*
		Mean ± SD	Median	Min, Max	Mean ± SD	Median	Min, Max		
Avg.wrinkle depth (μm)	Week 0	58.63 ± 14.040	55.0	39.0, 91.0	55.73 ± 9.915	57.5	36.0, 77.0	0.290	0.649
	Week 12	57.68 ± 14.543	53.5	35.0, 94.0	56.89 ± 10.752	58.0	33.0, 78.0	0.790	0.594
	Change	−0.95 ± 4.188	−1.0	−10.0, 7.0	1.51 ± 4.107	1.0	−6.0, 10.0	0.011 *	0.024 *
Wrinkle volume (mm^3^)	Week 0	7.48 ± 2.293	7.1	3.8, 13.1	7.16 ± 1.804	7.3	3.5, 10.2	0.483	0.766
	Week 12	6.87 ± 2.185	6.3	3.6, 12.5	6.78 ± 1.820	7.0	2.8, 10.0	0.838	0.768
	Change	−0.61 ± 0.583	−0.6	−2.2, 0.5	−0.28 ± 0.488	−0.4	−1.2, 0.9	0.009 **	0.017 *
Roughness area (μm)	Week 0	29.04 ± 6.516	27.4	18.7, 45.8	27.84 ± 4.834	28.2	19.1, 39.1	0.354	0.785
	Week 12	28.07 ± 6.686	26.3	17.4, 45.9	28.14 ± 5.133	27.3	16.7, 38.4	0.956	0.506
	Change	−0.97 ± 1.948	−1.0	−4.4, 3.7	0.43 ± 2.133	−0.1	−3.1, 5.0	0.004 **	0.009 **
Mean depth biggest wrinkle (μm)	Week 0	92.80 ± 38.260	89.0	33.0, 202.0	89.93 ± 38.254	82.5	38.0, 217.0	0.738	0.578
	Week 12	90.53 ± 37.694	90.0	35.0, 206.0	89.43 ± 37.946	81.0	39.0, 223.0	0.900	0.867
	Change	−2.28 ± 9.498	−1.5	−24.0, 21.0	−0.76 ± 10.145	1.0	−33.0, 18.0	0.500	0.259
Max depth biggest wrinkle (μm)	Week 0	352.35 ± 222.127	327.0	83.0, 1115.0	308.05 ± 155.214	255.5	101.0, 694.0	0.390	0.490
	Week 12	333.73 ± 201.506	295.5	87.0, 963.0	308.54 ± 170.860	257.0	103.0, 870.0	0.558	0.655
	Change	−18.63 ± 51.793	−12.5	−229.0, 86.0	−2.81 ± 62.583	−3.0	−164.0, 215.0	0.230	0.299
Wrinkle area (mm^2^)	Week 0	126.62 ± 18.148	135.6	85.6, 145.0	127.24 ± 17.042	135.4	93.6, 145.1	0.875	0.927
	Week 12	118.30 ± 16.146	126.5	82.5, 139.3	117.53 ± 16.524	125.2	86.8, 136.7	0.837	0.895
	Change	−8.31 ± 4.059	−7.0	−18.0, −2.4	−8.69 ± 3.335	−7.8	−19.0, −3.5	0.661	0.423
Length of wrinkle (mm)	Week 0	192.28 ± 35.695	201.5	118.0, 253.0	201.68 ± 26.586	203.5	143.0, 264.0	0.186	0.336
	Week 12	176.58 ± 30.836	184.5	113.0, 229.0	183.30 ± 25.500	188.0	133.0, 234.0	0.303	0.453
	Change	−15.70 ± 10.920	−16.0	−42.0, 11.0	−16.73 ± 10.090	−17.0	−39.0, 10.0	0.669	0.811

*p* < 0.05 *, *p* < 0.01 **, ^#^: Independent *t*-test, ^##^: Wilcoxon’s rank sum test.

**Table 3:** Changes from baseline value of biomarkers in skin.

Variable	Group	Week 0	Week 4	Week 8	Week 12	Significance	
						Factor	*p*
Skin Hydration (A.U.)	PTE	41.44 ± 5.86	42.14 ± 6.20	43.18 ± 6.40	44.34 ± 6.67	Group ^##^	0.772
	Placebo	41.42 ± 4.56	41.42 ± 4.91	41.88 ± 5.04	42.91 ± 5.35	Time ^†^	0.001 ***
	*p* for group ^#^	0.793	0.627	0.338	0.306	Group × Time ^‡^	0.057
Skin Elasticity (%)	PTE	74.41 ± 5.22	74.81 ± 5.15	75.40 ± 5.24	74.81 ± 5.31	Group ^##^	0.300
	Placebo	75.69 ± 4.33	76.12 ± 4.08	76.17 ± 4.33	76.29 ± 4.57	Time ^†^	0.003 ***
	*P* for group ^#^	0.240	0.221	0.487	0.196	Group × Time ^‡^	0.170
Skin glowing (GU)	PTE	3.37 ± 0.97	3.76 ± 0.98	3.86 ± 1.01	3.90 ± 1.02	Group ^##^	0.094
	Placebo	3.41 ± 0.92	3.44 ± 0.80	3.46 ± 0.86	3.55 ± 0.87	Time ^†^	0.001 ***
	*P* for group ^#^	0.154	0.129	0.066	0.111	Group × Time ^‡^	0.486
Transepidermal water loss (g/h/m^2^)	PTE	15.09 ± 4.14	14.78 ± 3.77	15.10 ± 4.83	14.64 ± 3.61	Group ^##^	0.0.211
	Placebo	16.25 ± 4.48	16.19 ± 3.64	15.44 ± 3.10	15.34 ± 2.69	Time ^†^	0.114
	*P* for group ^#^	0.233	0.101	0.710	0.346	Group × Time ^‡^	0.227

*p* < 0.001 ***. ^#^: *t*-Test of between groups. ^##^: between the placebo group and treatment group with repeated measure ANOVA. ^†^: within groups according to the duration of the trial with repeated measure ANOVA. ^‡^: *p* value for interaction between time and group by ANOVA for repeated measurements (time × group) between the placebo and the treatment.

**Table 4:** Survey results on product efficacy.

Variable	Group	Week 4	Week 8	Week 12	Significance	
					Factor	*p*
Increase skin moisture	PTE	3.33 ± 0.57	3.63 ± 0.62	3.63 ± 0.58	Group ^##^	0.599
	Placebo	3.22 ± 0.63	3.65 ± 0.48	3.54 ± 0.65	Time ^†^	0.001 ***
	*p* for group ^#^	0.430	0.855	0.550	Group × Time ^‡^	0.620
Increase skin gloss	PTE	3.20 ± 0.56	3.63 ± 0.58	3.60 ± 0.67	Group ^##^	0.182
	Placebo	3.14 ± 0.48	3.41 ± 0.49	3.46 ± 0.69	Time ^†^	0.001 ***
	*P* for group ^#^	0.590	0.082	0.369	Group × Time ^‡^	0.563
Increase skin elacsticity	PTE	3.25 ± 0.58	3.55 ± 0.55	3.65 ± 0.70	Group ^##^	0.066
	Placebo	3.22 ± 0.58	3.27 ± 0.45	3.38 ± 0.59	Time ^†^	0.001 ***
	*P* for group ^#^	0.801	0.018 *	0.071	Group × Time ^‡^	0.151
Improvement crow’s feet	PTE	3.05 ± 0.50	3.25 ± 0.49	3.65 ± 0.58	Group ^##^	0.337
	Placebo	3.11 ± 0.39	3.22 ± 0.53	3.35 ± 0.67	Time^†^	0.001 ***
	*P* for group ^#^	0.5770.	0.744	0.040 *	Group × Time ^‡^	0.025 *
Improvement total skin condition	PTE	3.40 ± 0.54	3.68 ± 0.52	3.90 ± 0.59	Group ^##^	0.138
	Placebo	3.32 ± 0.62	3.62 ± 0.54	3.57 ± 0.64	Time ^†^	0.001 ***
	*P* for group ^#^	0.573	0.663	0.021 *	Group × Time ^‡^	0.106

*p* < 0.05 *, *p* < 0.001 ***. ^#^: *t*-Test of between groups, ^##^: between the placebo group and treatment group with repeated-measure ANOVA. ^†^: within groups according to the duration of the trial with repeated-measure ANOVA. ^‡^: *p* value for interaction between time and group by ANOVA for repeated measurements (time × group) between the placebo and the treatment.

## Data Availability

Not applicable.
